# Detection and localization of multiple rate changes in Poisson spike trains

**DOI:** 10.1186/1471-2202-12-S1-P268

**Published:** 2011-07-18

**Authors:** Marietta Tillmann, Michael Messer, Markus Bingmer, Julia Schiemann, Ralph Neininger, Jochen Roeper, Gaby Schneider

**Affiliations:** 1 Institute of Mathematics, Goethe-University Frankfurt, Germany; 2 Institute of Neurophysiology, Neuroscience Center, Goethe-University Frankfurt, Germany

## 

In statistical spike train analysis, stochastic point process models usually assume stationarity, in particular that the underlying spike train shows a constant firing rate (e.g. [1]). However, such models can lead to misinterpretation of the associated tests if the assumption of rate stationarity is not met (e.g. [2]). Therefore, the analysis of nonstationary data requires that rate changes can be located as precisely as possible. However, present statistical methods focus on rejecting the null hypothesis of stationarity without explicitly locating the change point(s) (e.g. [3]).

We propose a test for stationarity of a given spike train that can also be used to estimate the change points in the firing rate. Assuming a Poisson process with piecewise constant firing rate, we propose a Step-Filter-Test (SFT) which can work simultaneously in different time scales, accounting for the high variety of firing patterns in experimental spike trains. Formally, we compare the numbers N1=N1(t,h) and N2=N2(t,h) of spikes in the time intervals (t-h,t] and (h,t+h]. By varying t within a fine time lattice and simultaneously varying the interval length h, we obtain a multivariate statistic D(h,t):=(N1-N2)/√(N1+N2), for which we prove asymptotic multivariate normality under homogeneity. From this a practical, graphical device to spot changes of the firing rate is constructed.

**Figure 1:**
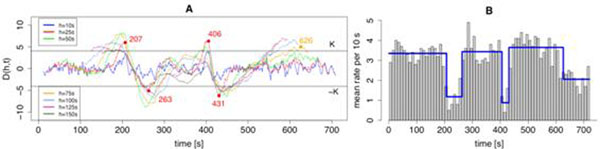
Graphical representation of the step filter test (SFT) that detects rate changes in Poisson spike trains. **A**. Curves indicate values of D(h,t) as a function of time t for different window sizes h (in different colors). **B**. Rate histogram (grey) of the corresponding spike train. Blue step function indicates estimated rate profile.

Our graphical representation of D(h,t) (Figure 1A) visualizes the changes in the firing rate. For the statistical test, a threshold K is chosen such that under homogeneity, |D(h,t)|<K holds for all investigated h and t with probability 0.95. This threshold can indicate potential change points in order to estimate the inhomogeneous rate profile (Figure 1B). The SFT is applied to a sample data set of spontaneous single unit activity recorded from the substantia nigra of anesthetized mice. In this data set, multiple rate changes are identified which agree closely with visual inspection. In contrast to approaches choosing one fixed kernel width [4], our method has advantages in the flexibility of h.
